# The Use of Mobile Health Technology and Behavioral Economics to Encourage Adherence to Statins and Blood Pressure–Lowering Medication in Adolescents with Familial Hypercholesterolemia or Hypertension: Protocol for a Pre-Post Cohort Study

**DOI:** 10.2196/65105

**Published:** 2025-08-14

**Authors:** Jacob Hartz, Hannah Chiert, Sarah de Ferranti, Tiffany Powell-Wiley

**Affiliations:** 1 Department of Cardiology Boston Children's Hospital Boston, MA United States; 2 Department of Pediatrics Harvard Medical School Boston, MA United States; 3 Social Determinants of Obesity and Cardiovascular Risk Laboratory Cardiovascular Branch, Division of Intramural Research National Heart, Lung, and Blood Institute, National Institutes of Health Bethesda, MD United States; 4 Intramural Research Program, National Institute on Minority Health and Health Disparities National Institutes of Health Bethesda, MD United States

**Keywords:** dyslipidemia, hypertension, adherence, mobile health, incentives, behavioral health, youth

## Abstract

**Background:**

Cardiovascular disease (CVD) is a leading cause of mortality and morbidity in the United States, with risk factors such as hypertension and elevated low-density lipoprotein (LDL) cholesterol originating in childhood. While statins and blood pressure–lowering medications can mitigate these risks, adherence is often poor, particularly among youth. Innovative solutions, such as monetary incentives via smartphone apps, may enhance adherence, but evidence in youth is lacking.

**Objective:**

This study aims to evaluate the efficacy of a smartphone app (Wellth) offering financial incentives to improve adherence to statins and blood pressure–lowering medications among youth aged 12 to 19 years at risk for cardiovascular disease.

**Methods:**

We initially designed a randomized controlled trial to compare the efficacy of 2 different incentive structures in youth treated with a statin for familial hypercholesterolemia with inadequate adherence. After facing recruitment challenges, the study protocol was changed to evaluating a single incentive in a pre-post design. The primary outcome was the change in adherence rate over the 60-day incentive period compared to the adherence rate during the 14-day run-in period. The secondary outcome was a change in LDL cholesterol level. Adjustments to the protocol were made in response to recruitment challenges during the COVID-19 pandemic, simplifying the incentive structure and expanding eligibility criteria.

**Results:**

The study is currently undergoing recruitment and collection of data from the first participants. The study has faced recruitment challenges exacerbated by the COVID-19 pandemic, necessitating protocol modifications. Detailed analysis of adherence rates and LDL cholesterol changes is ongoing.

**Conclusions:**

This study explores the efficacy of monetary incentives delivered through a smartphone app to improve medication adherence in youth at risk for CVD. The findings will be used to build upon the existing literature in an effort to improve medication adherence throughout the life course and ultimately reduce CVD.

**Trial Registration:**

ClinicalTrials.gov NCT04458766; https://clinicaltrials.gov/study/NCT04458766

**International Registered Report Identifier (IRRID):**

DERR1-10.2196/65105

## Introduction

Cardiovascular disease (CVD) is the leading cause of death and disability in the United States. It leads to US $216 billion in direct costs and an additional US $147 billion in indirect costs each year [1]. While the presentation of CVD events primarily occurs in adulthood, the significant risk factors for CVD, including hypertension and elevated low-density lipoprotein (LDL) cholesterol, have their antecedents in childhood [2,3]. With the use of statins [4-17] and blood pressure–lowering medications [18], substantial reductions in this risk are possible.

However, adherence to statins and blood pressure–lowering medications is needed to accrue these potential benefits. Unfortunately, medication adherence is frequently poor, with up to 50% of adults not taking all their prescribed medications [19-23]. In adults, nonadherence is associated with an increased risk for CVD events and all-cause mortality [20,24], as well as almost US $44 billion annually in additional health care costs [25,26]. Although studies are limited, adherence to statins and blood pressure–lowering medications also has been shown to be inadequate in youth [27-30]. Youth seem to be particularly vulnerable to nonadherence [31], even in conditions in which the consequences of nonadherence can lead to rapid disease progression or severe, acute symptoms, such as bipolar disorder [32], HIV infection [33], or asthma [34].

**Figure 1:**
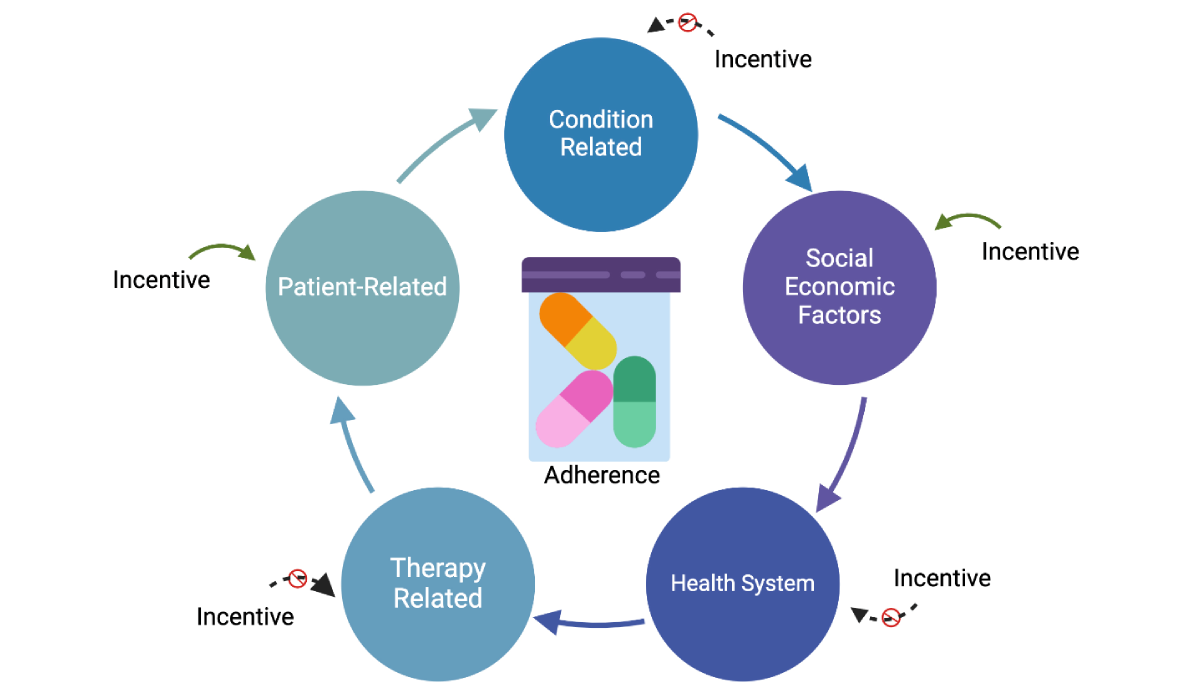
Five components of medication adherence as proposed by the World Health Organization [35]. The green curved arrows indicate components expected to respond to incentives, while the dashed black arrows are components that are less likely to respond.

Multiple factors impact adherence (Figure 1), including patient-related factors (eg, beliefs and expectations), socioeconomic factors, medication-related factors (eg, complexity of the regimen and adverse effects), the disease being treated, and the health care system [35]. Unfortunately, successful interventions to improve medication adherence overall are rare in both adults [36] and youth [36,37]. Further, there are no formally tested interventions to improve adherence to statins or blood pressure–lowering medications in youth. One potential solution to improve adherence is to provide monetary incentives. Traditional economic theory suggests that providing individuals with incentives to complete an action will lead to increased frequency of the occurrence of the desired action. Studies in adults have shown monetary incentives to be modestly successful in certain diseases [38-40], but not all studies have shown positive results [41]. Further, it is unclear if these findings translate to youth.

To address this gap, we planned a study using a smartphone app (Wellth) to provide financial incentives to improve medication adherence in youth at risk of CVD. Smartphone apps are promising interventional tools as they are familiar to most youth [42,43]. Data suggests using smartphone apps in a health care setting is of interest to youth [44] across a diverse set of socioeconomic and cultural backgrounds [42]. The Wellth app is designed to improve medication adherence through reminders and by providing monetary rewards for taking a medication as prescribed.

The objective of this paper is to describe the original study design, recruitment methods, outcomes, analysis plan, strengths, and limitations. In addition, we highlight modifications to the design and recruitment strategies because of social distancing requirements related to the COVID-19 pandemic and the need to improve recruitment. The principal aim of our study is to determine if financial incentives are effective at improving adherence to medications in youth who are at an increased risk of CVD.

## Methods

### Study Design

**Figure 2:**
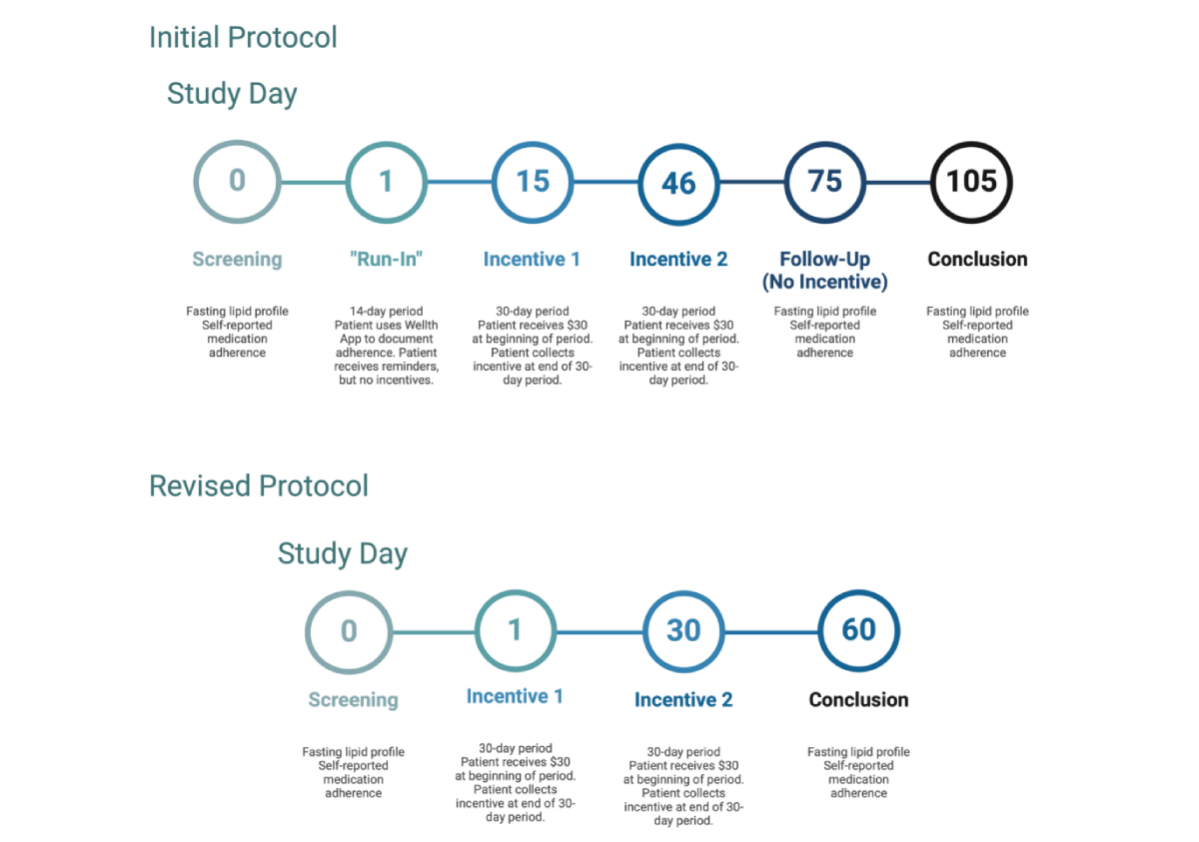
Final study timeline. The fasting lipid profile included total cholesterol, triglyceride level, high-density lipoprotein cholesterol, and low-density lipoprotein cholesterol.

The study, originally titled “Use of Mobile Health Technology and Behavioral Economics to Encourage Adherence to Blood Pressure–Lowering Medications and Statins in Youth at Risk for Cardiovascular Disease,” was designed as a randomized control trial to compare the efficacy of 2 interventions on improving medication adherence to statins in youth aged 12 to 19 years using a smartphone app (Figure 2). The 104-day study consisted of a 14-day run-in period, 2 periods of 30 days in which participants received incentives, and a 30-day follow-up period in which participants were asked to record adherence but did not receive any incentives. During the run-in and follow-up periods, all features of the Wellth app were available, but the participants did not receive any monetary incentives. This approach was used to isolate the effect of the monetary incentive from the other features of the app and to provide an objective baseline measure of adherence. After the 14-day run-in, the participants were eligible to receive incentives during two 30-day incentive periods. Incentives were disbursed at the end of period 1 (day 44) and period 2 (day 74). In addition, we also measured the patients’ adherence in the 30-day period after the incentive periods to determine if the effect of the intervention was sustained. The study was approved by the research ethics board at the Boston Children’s Hospital with a waiver of individual participant consent as this research used data that had previously been obtained through a departmental quality improvement project. Patients who were older than 18 years at any time while participating in the study signed an informed consent form. For patients who were younger than 18 years, informed consent was obtained from their legal guardian, and assent was obtained from the participant.

### Eligibility Criteria

The original eligibility criteria are described in Textbox 1. Patients were eligible to participate if they were aged 12 to 19 years, had a diagnosis of familial hypercholesterolemia (FH), were prescribed a statin, and reported less-than-ideal statin adherence. The diagnosis of FH was based on genetic testing or if the patient met the National Lipid Association recommendations, which are an LDL cholesterol level greater than or equal to 160 mg/dL or a non–high density lipoprotein cholesterol level greater than or equal to 190 mg/dL in patients younger than 20 years [11,19,20,45].

**Eligibility and exclusion criteria.**

**Eligibility criteria**

- Less-than-ideal adherence by self-report
- Age 12 to 19 years
- Diagnosis of familial hypercholesterolemia (FH) based on National Lipid Association criteria and/or genetic testing or a diagnosis of hypertension
- Prescribed a statin or blood pressure–lowering medication
- Ability to provide written informed consent or have a parent or guardian provide written informed consent

**Exclusion criteria**

- Homozygous FH
- Residence in a long-term care facility where medications are administered
- Being pregnant/possibility of becoming pregnant
- History of adverse effects or allergies to a statin or blood–pressuring lowering medication or any ingredient in one of these medications

### Recruitment

We recruited patients from a tertiary care hospital and its satellite outpatient clinics. Recruitment took place at outpatient clinic visits in a preventive cardiology clinic. The study clinicians approached eligible patients and provided a brief overview of the study. If the participant was interested, the clinician determined the patient’s eligibility based on self-reported adherence [46,47]. If the patient was eligible and agreed to participate, the clinician conducted the informed consent and enrollment procedures.

We supplemented clinic recruitment by recruiting patients with FH treated with a statin by phone using a database maintained by the preventive cardiology program, which includes approximately 400 patients. We estimated that 20 patients per month who met the eligibility criteria would be seen. If 50 percent of those patients agreed, we expected to complete the recruitment of 30 patients in 6 months (power calculation shown below). Patients could be removed from the study if there was concern for their safety or there was a change in the medication that they were prescribed. Patients could voluntarily stop participating in the study at any time.

### Outcomes

The primary outcome was medication adherence as measured by the Wellth app and defined as the proportion of doses taken per doses prescribed. The adherence rate used in the analysis was measured over one 14-day period and three 30-day periods (days 1-14, days 15-44, days 45-74, and days 75-104). Secondary outcomes included change in the LDL cholesterol level from baseline until the end of the intervention period (day 74). LDL cholesterol was considered a baseline measurement if it was obtained within 60 days from the day of consenting to the study. The covariates we included in the analysis were gender, age, and race/ethnicity.

### Description of Wellth App

The Wellth app was the primary tool used in the study to promote medication adherence. Patients used the app to receive reminders, make check-ins (ie, document adherence), check their reward balance, and view their adherence history. Participants downloaded the app to their smartphone without any additional software or encryption needed. In order to prevent patients from being excluded based on the costs of the necessary hardware, we provided a smartphone and data plan free of charge to patients. Wellth monitored app use and uploaded photos through a secure, customized analytics dashboard for data monitoring and customer support.

### Structure of Incentives

Initially, participants were to be randomized to 1 of 2 incentive structures. The first structure of the incentive was based on the principles of present bias and loss aversion. Present bias refers to the tendency of individuals to prefer more immediate rewards and outcomes and to discount future risks [48]. For instance, studies suggest that even small rewards, if provided frequently and immediately after a participant completes a desired task, can improve adherence in adults and may be more effective at promoting behavior change than larger rewards provided in the distant future [36,49,50]. The principle of loss aversion was included to help strengthen the incentive and is based on findings from studies that suggest individuals place more value on money or objects that they have than on new objects of the same value [51].

Participants received US $30 at the beginning of each of the two 30-day periods but were only able to access the money at the end of the 30-day periods. However, participants could view their balance within the app at any time. For each missed check-in, the participant lost US $2 from the amount to be paid out at the end of the 30-day period. Participants who missed more than 15 check-ins did not receive any money at the end of the 30-day period.

In the second incentive approach, we planned for participants to not receive rewards for each check-in but instead receive the *chance* to receive a reward of varying amounts. This incentive structure is similar to that of a slot machine. As in the first incentive structure, the total amount a participant could receive over the two 30-day periods was US $60. However, participants would not lose money if a check-in was missed; rather, they would lose an opportunity to receive a reward. The random distribution of the reward was structured to ensure that a fully adherent participant would receive a full US $60 over the study period. However, in this arm of the study, it was also possible that a participant could receive US $60 even if they were not fully adherent.

### Power

Power calculations were planned to be performed for the primary outcome variable of adherence rate at the end of the 60-day period. The mean difference in adherence rates at 60 days after the start of the intervention (day 74) would be compared to 0%, which was the value that would be expected if there was no difference between the two interventions using a 2-sided paired *t* test conducted at the .05 level of significance. The mean adherence rate was hypothesized to be 80% when patients received small frequent awards and 74% when receiving a large, randomly delivered reward, suggesting a mean within-patient difference of 6%. The SD of the adherence rate was assumed to be 10% at each time point [27], and the correlation between the two measures of adherence was assumed to be 0.50, resulting in an SD of differences in adherence rate of 10%. If the correlation between measurements of adherence was conservatively assumed to be 0.30, the SD of differences in adherence rate was estimated to be 11.8%. We expected to enroll 30 patients.

### Statistical Analysis

Data were planned to be analyzed on an intention-to-treat basis. Baseline patient characteristics measured prior to randomization would be compared between the two intervention groups to look for imbalances. Primary and secondary outcomes would be compared between groups on day 44, day 74, and day 104. Continuous variables would be compared using the 2-sample *t* test or Wilcoxon rank sum test. Differences in categorical variables would be assessed using the Fisher exact test. Analyses of the primary outcome variables (ie, percentage change in LDL cholesterol and proportion of days adherent) would use a significance level of .025 at each follow-up time point; all other comparisons would be performed at the .05 level of significance. If differences in baseline characteristics were detected between the two groups, linear regression would be used to perform additional comparisons of the primary and secondary outcomes, controlling for these potential confounders. Transformations would be applied to continuous outcomes that were not normally distributed.

### Ethical Considerations

This study was approved by the Boston Children's Hospital Institutional Review Board (IRB-A00032841-2).

A majority of participants in the proposal are younger than 21 years and thus meet the National Institutes of Health’s definition of children. The study will not exclude participants based on race/ethnicity, gender, or sexual preference. The final study sample will be representative of the patient population of the Boston Children’s Hospital Preventive Cardiology Program. Our sampling plan was developed in consultation with a statistician to ensure appropriate representation of all groups.

Privacy is a fundamental concern any time that personal information is transmitted electronically, especially if a third party is involved. Wellth does not share patient data with any third party. Wellth uses deidentified datasets wherever possible, including internal reports and communications. Wellth will share data with members of the Boston Children’s Hospital study team needed for data analysis and patient safety monitoring. Wellth uses HITRUST (Health Information Trust Alliance)-certified, HIPAA (Health Insurance Portability and Accountability Act)-compliant application hosting services provided by HealthcareBlocks. All computers used by Wellth are encrypted, including hosting computers. Every member of the company performs annual HIPAA compliance training. All software development and company activities are performed inside the United States. We specifically instructed participants not to upload photos that include any personally identifying information.

## Results

The intervention was developed to encourage patients to take their medication every day, and the pre-post study was used to determine the efficacy of this intervention. The findings of the study will help determine if monetary incentives can lead to increased adherence and help determine if this is a viable strategy that can be expanded. Recruitment started in August 2019 and concluded in August 2024 (n=9 participants were recruited). Data collection was ongoing throughout the trial and will be analyzed after study completion. The study’s findings are to be published after study completion, which is expected in December 2025 or January 2026.

## Discussion

### Anticipated Findings

We expect to find that providing small, frequent financial incentives will improve medication adherence among youth taking medications to reduce their risk for cardiovascular disease. We expect that this improved adherence will be demonstrated by an increase in adherence as measured by the Wellth app.

Unfortunately, only a few studies with adolescents have used incentives to promote behavioral change. In a study with youth with type 1 diabetes, incentives had a positive impact on glucose monitoring frequency and adherence to the prescribed insulin regimen [50]. However, patients with diabetes mellitus fundamentally differ from those with hypertension or dyslipidemia, as the latter conditions are typically asymptomatic [7]. While studies in adults have had mixed results [52], adolescents differ substantially regarding attitudes toward health, and they have less mature cognitive function and capacities than adults [49].

### COVID-19 Pandemic

Our initial protocol and recruitment strategy had to be modified in response to the challenges that arose during the COVID-19 pandemic. In the original proposal, we planned to recruit patients seen during routine outpatient visits. However, in the early months of the pandemic, outpatient clinic appointments were limited to those with urgent concerns, which rarely included patients with dyslipidemia. Enrolling participants remotely, though, posed several challenges. The first was connecting the patient to our research assistant. Unlike an in-person clinic visit in which the research assistant is readily available, connecting the research assistant with the patient during a remote encounter required arranging additional contact with the patient. We found that if there were any delays in connecting to the research assistant, eligible participants seemed to quickly lose interest.

A second problem arose in obtaining written consent from participants seen via telehealth visits. Unfortunately, the infrastructure for obtaining an electronic signature was initially unavailable and written consent was required by our institution. The written consent was rarely completed during the course of a virtual visit. Instead, families typically planned to send the signed consent forms that evening or the next day, which often did not occur despite repeated attempts to contact eligible patients. In addition, errors in the consent, such as missing signatures or illegible handwriting, often led to patients not participating, as obtaining corrected consent forms was challenging.

The third barrier that arose was related to technical issues signing patients up for the Wellth app. While configuring the app for the patient was not cumbersome, and there were no complaints by participants enrolling in the study, adding study-related information (eg, participant identification number and study codes) was sometimes technically challenging. We could not have the participant download the smartphone app until the consent was finalized, which often meant arranging a separate time to ensure the app was operational, and some participants dropped out after signing the consent form because of the additional time commitment.

### Low Recruitment

Recruitment for the trial was more difficult than expected, in part related to the COVID-19 pandemic. We were concerned that the complexity and duration of the trial were deterrents. Over the first year, we made a series of changes to simplify the intervention.

First, we decided to use only the first intervention, which involved small, frequent rewards, while removing the second intervention, which entailed random, variable rewards. This was based on our perception that the first intervention was conceptually easier for pediatric participants to understand. Since we would no longer be comparing two types of incentive structures, we also removed randomization and created a pre-post study design (Figure 2). Adherence measures collected via the app during the 14-day run-in period would be used as the baseline adherence measure to be compared to adherence recorded on day 44, day 74, and day 104.

The second modification was to shorten the trial duration by removing the 30-day follow-up period. We received feedback from potential participants during recruitment that the length of the trial prevented them from participating. As this portion of the trial was included mainly to assess the sustainability of the incentive, it made more sense to remove it rather than shorten the incentive period.

Third, we expanded the protocol to include patients with hypertension who had been prescribed a blood pressure–lowering medication (eg, angiotensin-converting enzyme inhibitors, angiotensin receptor blockers, diuretics, or calcium-channel blockers) to increase the pool of eligible participants. We did not change the incentive structure, even for those patients who were prescribed regimens with twice-daily dosing. In these patients, we required both doses to be taken to earn the reward. The diagnosis of hypertension was based on chart review (ie, hypertension-associated diagnosis codes in the *International Classification of Diseases*, ninth or tenth revisions) and was not based on blood pressure–specific thresholds.

### Limitations

As has been acknowledged, recruiting for pediatric studies can be difficult. To overcome this obstacle, we changed the protocol and recruitment strategies. In the future, we plan to increase the pool of recruits by expanding to other clinics outside of Boston Children’s Hospital. Another potential limitation is the inability to ensure that the increase in adherence is specifically from the incentives and not another component of the Wellth app. We have tried to mitigate this potential problem by not offering the incentive for the first two weeks. We will compare the adherence during the first two weeks with adherence during the periods when incentives are provided to isolate the effect of the incentive.

### Future Directions

This research protocol will test whether providing incentives that leverage the principles of behavioral economics can improve medication adherence in youth with FH and hypertension. Understanding the effectiveness of different types of incentives is crucial to improve medication adherence. The incentive structure in regard to the type of reward, frequency of receiving the award, and timing of delivery of the reward can lead to different responses. In fact, there is evidence that inappropriate reward structures can serve as a negative enforcer.

In addition, this proposal will harness the power of digital health technology, taking advantage of the fully developed Wellth app to reduce costs and simplify the intervention. Digital health apps allow for automating patient–health care provider interactions, improving monitoring using cameras with embedded metadata (eg, date and time), and reducing the complexity of interventions, allowing for more widespread distribution of interventions.

Patients with FH and hypertension provide an ideal cohort to explore the role of different incentive packages. FH is rarely symptomatic in youth, and treatment is typically well tolerated. One critical factor is that missed doses do not generally lead to acute complications in either disease. Therefore, health care providers and caretakers can safely distance themselves from interfering, which prevents adding biases introduced by frequent health care provider engagement.

It should be acknowledged that there are important differences between those prescribed lipid-lowering therapy for an inherited condition and those treated for hypertension, which is typically an acquired condition. Further, blood pressure–lowering medications have a different side effect profile than statins and may be prescribed more than once daily, which may impact adherence.

### Conclusion

This proposal will elicit information critical for creating developmentally appropriate incentive structures to improve medication adherence in youth with risk factors for CVD. However, the efficacy of monetary incentives is extrapolated from adult studies, and it remains to be seen if they are ideal for youth.

## Appendix

Multimedia Appendix 1 Peer review report form the HLBI Mentored Patient-Oriented Research Review Committee - Heart, Lung, and Blood Initial Review Group MPOR (MA) - National Heart, Lung, and Blood Institute (National Institutes of Health, USA).

## Abbreviations

CVD: cardiovascular disease
FH: familial hypercholesterolemia
HIPAA: Health Insurance Portability and Accountability Act
HITRUST: Health Information Trust Alliance
LDL: low density lipoprotein

## Data Availability

The datasets used and analyzed in this study are available from the corresponding author on reasonable request.
