# Supplementary material for: The Use of Mobile Health Technology and Behavioral Economics to Encourage Adherence to Statins and Blood Pressure–Lowering Medication in Adolescents with Familial Hypercholesterolemia or Hypertension: Protocol for a Pre-Post Cohort Study
Source: JMIR Res Protoc. 2025 Aug 14;14:e65105. doi: 10.2196/65105 (PMC12395099; doi:10.2196/65105)
Supplement: Multimedia Appendix 1 [file resprot_v14i1e65105_app1.pdf]

1 K23 HL 145109-01A1 HARTZ, JACOB

RESUME AND SUMMARY OF DISCUSSION: This is a K23 resubmission from Dr. Jacob Hartz in which he proposes a plan for his career development as an independent investigator focused on the development of mhealth interventions based on behavioral economic principles to improve medication adherence. The Candidate has been very responsive to the previous review concerns in many aspects. His Career Development Plan is considerably improved, now including training in clinical trial design and implementation, clearly defined metrics for progress, and a timeline. A remaining concern is the Candidate's overall publication record. While he lists several completed manuscripts, at the time of review they were not yet published. The Research Plan addresses an important clinical problem and the use of behavioral economic principles is innovative, but the research proposed is exploratory does not address an hypothesis and is not supported on preliminary findings. The previous review noted that the aims were interdependent, and this remains a concern. The mentoring team has the representative complementary expertise, but the primary mentor's lack of R01 funding is a concern, as is the lack of K level mentoring experience. Overall, this resubmission is significantly improved but there are a few remaining weaknesses.

DESCRIPTION (provided by applicant): Familial hypercholesterolemia (FH) affects over one million Americans and increases the risk of cardiovascular disease (CVD) by as much as 20-fold. Although the use of statins can substantially reduce this risk, adherence to statins in adults and adolescence is poor. In adults, lower rates of adherence are associated with an increased rate of CVD events and all-cause mortality, as well as an additional $44 billion annually in health care costs. Novel interventions are needed to improve medication adherence in patients with FH, starting in adolescents. An underused strategy to improve medication adherence incorporates the principles of behavioral economics. Traditional economic theory suggests that providing an incentive to perform a behavior will increase the frequency of that behavior. [However, two prominent theories in behavioral economics, Present Bias and Loss Aversion, suggest that not all types of incentives are effective and that poorly structured incentives can actually be negative enforcers. With novel mobile health technologies (mHealth), interventions based on behavioral economics can now be studied on a larger scale. In this proposal, the candidate 1) will test the efficacy of an mHealth intervention based on Present Bias and Loss Aversion to improve statin adherence in adolescents with FH in a randomized crossover trial; 2) gain a better understanding of the barriers to adherence in adolescents with risk factors for CVD using focus groups; and 3) perform a cost- effectiveness analysis with two-way sensitivity analysis of the interventions in Aim 1.] To achieve these goals and prepare for independence, a comprehensive career development program has been developed to gain additional training in: 1) clinical trials, 2) mHealth, 3) qualitative methods, 4) behavioral economics, and 5) cost- effectiveness analysis. Along with formal coursework, the candidate's mentoring and advisory committee includes experts in FH and lifestyle interventions (Sarah de Ferranti), mHealth in CVD (Tiffany Powell-Wiley), cost-effectiveness analysis (Mihail Samnaliev), biostatistics and clinical trials (Kimberlee Gauvreau), internationally-known leaders in pediatric cardiology and clinical trials (Jane Newburger), and behavioral economics (Jason Doctor). Boston Children's Hospital, Harvard School of Public Health, and the National Heart, Lung, and Blood Institute provide unparalleled environments to prepare a candidate for independence. Ultimately, the candidate's long-term goals are to strengthen the understanding of factors that influence health behaviors in adolescence and to design cost-effective, personalized interventions to help reduce their risk for CVD. [The additional training and findings from this trial will be the basis for future R01 proposals that will expand this proposal to include multiple clinical sites with more diverse cohorts and to adolescents with other risk factors for CVD.]

PUBLIC HEALTH RELEVANCE:
Premature cardiovascular disease has safe and effective p
retreatments available, but poor medication adherence in adults and adolescents undermines its potential benefits. We seek to evaluate an innovative approach to improve medication adherence in

adolescents by combining the principles of behavioral economics with novel mobile health technologies. Effective mobile technologies and behavioral economics have vast potential to modify lifestyle behaviors across a variety of conditions and reduce the risk of cardiovascular disease in a cost-effective manner.

CRITIQUE 1:

Candidate: 2
Career Development Plan/Career Goals /Plan to Provide Mentoring: 2
Research Plan: 4
Mentor(s), Co-Mentor(s), Consultant(s), Collaborator(s): 2
Environment Commitment to the Candidate: 2

Overall Impact:

This is a revised K23 application from a candidate who is an Instructor of Pediatrics with a clinical and research interest in Preventive Cardiology. The candidate is currently supported by a T32 and overall is a strong candidate with an excellent educational and training background with good early productivity for career level. Publications are primarily in areas related to this proposal. The career development plan is improved from the prior submission and has addressed many of the concerns raised. Increased training in the area of clinical trial design and implementation as well as the addition of more clear metrics for training goals and a more detailed timeline. The plan has some aspects that may be overambitious, but this is a minor weakness. The clinical problem addressed by this proposal is significant- non-adherence to medical therapy in an at risk group of adolescents and young adults. The design of Aim 1 (comparing the 2 different incentives in a cross-over design), the inclusion/exclusion criteria and the outcome measures chosen are improved from the prior submission. There still remains minor concerns in other areas, such as lack of preliminary data with the Wellth app, discrepancies throughout the application regarding the focus group numbers and composition, and details of how outcomes will be defined for the cost-effectiveness analysis in Aim 3 are not clear. Overall, these are relatively minor weaknesses, but deserve consideration in the implementation of the project. The research experience and content expertise of the primary co-mentors, who are both excellent clinical investigators are a strength of the proposal. Some concern regarding the inexperience in the mentors successful track record in serving as primary K mentors, and success in transitioning a candidate from a K to an R. The environment is outstanding with excellent research infrastructure and appropriate patient population with easy access for the candidate. The overall impact of the proposal is high, but minor weaknesses in the research plan remain the primary area of concern.

1. Candidate:

Strengths

- Excellent educational and training background, appropriate for this career development mechanism of funding.
- Short and long term goals are clearly described and are consistent with the K23 mechanism.
- Good publication record for level of training (9 publications [5 original science], 7 first author).

Weaknesses

- No prior grants or research support, except the current T32 (minor weakness given career stage).

2. Career Development Plan/Career Goals & Objectives:

Strengths

- Appropriate courses emphasizing training in clinical trial development are included. This is an improvement from the prior submission.
- Meetings with mentors are mostly well described and timely (one exception commented on below).
- Training objectives are clearly described and appropriate for the candidate's background and future career plans.
- Metrics of accomplishment for each training objective are described and are reasonable and measurable.

Weaknesses

- Weekly one hour long meetings by phone, in addition to remote attendance at weekly lab meetings, with co-primary mentor at the NHLBI may not be practically feasible or realistic.

3. Research Plan:

Strengths

- Research addresses the ubiquitous problem of nonadherence in the adolescent population, and is therefore highly significant. Results of this study could be extrapolated to other adolescent medical populations, so potential impact is high.
- Study is highly significant and the scientific premise is strong as modulation of cardiovascular disease risk factors in childhood/adolescence has implications for adult health.
- Overall, the aims of the project are appropriate and have potential to inform future investigations, providing the framework for an R01.
- Studies of incentives in improving adherence have been somewhat neglected in the adolescent population and deserve focused study. The design of Aim #1, comparing 2 types of incentives in a cross-over trial design, is a strength of the study.
- Choice of patients for this study is supported by solid rationale.

Weaknesses

- Unclear where the 85% adherence cutoff for inclusion comes from.
- No preliminary data using the Wellth app is provided raising some concerns for feasibility.
- Rationale for assessing difference in adherence between interventions, and not between baseline and each intervention independently is not clear.
- Lack of detail for focus groups in the research plan and discrepancies throughout the application regarding the number of groups planned. There is no rationale provided for the number of focus groups needed for this study, and it is unclear what measures will be used to determine if more or less groups will be needed. There is some discussion regarding consideration of age for the make-up of the groups, but no discussion of considerations for sex or SES in forming the groups. No inclusion/exclusion criteria for caregivers.
- For the cost-effectiveness analysis it is not clear how the "treatment goals" are defined.

4. Mentor(s), Co-Mentor(s), Consultant(s), Collaborator(s):

Strengths

- Very good group of mentors assembled with complementary areas of expertise. The role of each mentor in the training process is clearly outlined and well supported by the research plan.

Weaknesses

- Limited information regarding mentee track record of primary and co-mentor. Unclear if either have been primary mentors on a K award in the past and success in mentoring an individual through the K to R transition is not stated (minor weakness). However, both are clearly outstanding investigators.

5. Environment and Institutional Commitment to the Candidate:

Strengths

- Candidate on a T32 with current 75% protected time for research demonstrating good institutional commitment to this candidate's career development.

Weaknesses

- Unclear if candidate's current faculty position is contingent on K23 funding; not explicitly stated. No startup funding provided.

Study Timeline:

Strengths

- Overall reasonable timeline that accounts for major components of the research plan.

Weaknesses

- Number of subjects to be enrolled by specific timepoints during the study is not included.

Protections for Human Subjects:

- Acceptable Risks and Adequate Protections
- Some discrepancy in age of subjects; Table 1 and 3 state 12-19 years, while the Human Subjects forms state age 13-19.

Resubmission:

- Prior review demonstrated concerns primarily with the career development plan and the research plan. There is an improved career development plan with more detail included regarding metrics of success throughout the project as suggested, additional structured training in clinical trial design and NIH based workshops. The research plan is also improved and addresses most of the issues raised with the prior review, but there remain several weaknesses. Specifically, the primary outcome for the interventions planned in Aim 1 is now adherence, with change in LDL a secondary endpoint. However, there are some areas in the application where LDL change is still listed as a primary endpoint. The concern for how age (wide range) could impact results for the Aim 1 clinical trial was not addressed. There is still no preliminary data raising some concerns for feasibility of using this specific app, discrepancies in the number and composition of focus groups, and lack of clarity regarding how data will be interpreted for Aim 3 are ongoing concerns.

CRITIQUE 2:

Candidate: 3
Career Development Plan/Career Goals /Plan to Provide Mentoring: 3
Research Plan: 6

Mentor(s), Co-Mentor(s), Consultant(s), Collaborator(s): 3
Environment Commitment to the Candidate: 3

Overall Impact:

This is a K23 resubmission from a talented and motivated junior faculty interested in economics of health and medication adherence. Modest publication record (although not unusual for this stage of training). Career development plan with attention to coursework on clinical trials, mobile health and behavioral economics, although limited in scope and does not address specifics (mobile app programming, focus group training). The research plan will look at a mobile health app (Wellth) and compare two different reward systems in motivating subjects to take cholesterol meds, perform focus groups on barriers to medication adherence and look at cost effectiveness. There is no over-arching hypothesis, but improvement in justification for behavioral economics interventions. Mentors do not describe extensive experience with K awardee mentoring, and primary mentor without record of NIH funding. Institution willing to maintain salary but does not discuss continued protection of applicant's research time. In summary, interesting proposal from motivated junior faculty but remaining weakness in research plan diminishes enthusiasm. Would encourage continued effort should this not be funded as applicant is clearly meritorious.

1. Candidate:

Strengths

- T32 training currently.
- 3 first author research manuscripts plus several review articles.
- Early background in economics.
- Master's in public health.

Weaknesses

- Modest publication record compared to completion of fellowship in 2017.
- Would be helpful if completed manuscripts were accepted for publication.
- No independent funding beyond T32.

2. Career Development Plan/Career Goals & Objectives:

Strengths

- Looking at use of behavioral economics and mHealth to look at effects of different incentives on medication adherence in adolescents.
- Planned coursework on clinical trials, mobile health, behavioral economics.

Weaknesses

- Training area 2, qualitative research methods, will learn focus groups from Dr. Revette, but no letter of support, no specifics on how this will be done and no specifics in research plan either.
- If applicant plans career with mobile health technologies, may wish to broaden education in that area rather than focus on this single app.
- Given economics background unclear how much more training in that area is relevant.

3. Research Plan:

Strengths

- Aim 1 looks at interventions to improve statin adherence.
- Aim 2 uses focus groups to look at barriers to medication adherence.
- Aim 3 looks at cost effectiveness of two different interventions from Aim 1.
- Innovative approach to adherence in a chronic disease.

Weaknesses

- More discovery than hypothesis driven.
- Major weakness is lack of preliminary data provided, which would be helpful in justifying power calculations and feasibility and lacking despite this being a resubmission.
- Logical progression would be Aim 2, then Aim 1 then Aim 3. Concerned that Aim 2 will yield suggestions that are not part of Aim 1, reducing the efficacy and utility of Aim 3
- Only examines two different incentive structures and no evidence presented that these are the key methods required in this age group.
- Preliminary data lacking for these aims-presented that adherence is poor, that adolescents have different barriers and that adults respond to the Wellth app, but no data on this particular population. Would like to see data that the applicant can enroll subjects, obtain compliance, and perhaps even prelim results with this type of app (cannot find published results with it). In addition, no data provided regarding feasibility, burden, and compliance, all of which will affect results.
- While power calculations are provided, there is no accounting for dropouts-I would find it difficult to assume that in this age group full and useable data sets would be available for all subjects.
- Minimal data provided on how the focus groups will work and what questions will be asked for Aim 2.
- Table 5 in recruitment and retention plan shows a timeline where Aim 2 is actually Aim 1, a strategy that makes more sense logically but also makes it unclear why applicant requires funding for year 5.

4. Mentor(s), Co-Mentor(s), Consultant(s), Collaborator(s):

Strengths

- Dr. de Ferranti well suited to proposal, with history of K awardee mentoring, director of preventative cardiology, and comparative effectiveness of cholesterol screening.
- Dr. Powell-Wiley also with excellent background for this research and well-funded
- Dr. Samnaliev with health economic background to help with cost effectiveness analysis.

Weaknesses

- Primary mentor Dr. de Ferranti with no current funding other than co-PI role and no listed R01.
- Mentors do not detail any history of mentoring K23 recipients in their letters except for Dr. de Ferranti serving as a co-mentor.
- Letters of support from Wellth suggest their platform will support Aim 2 but per research plan this appears to be used in Aim 1? Suspect due to switch in order of aims.

5. Environment and Institutional Commitment to the Candidate:

Strengths

- Good institution with good cardiology program.

Weaknesses

- Institution letter says “salary will be maintained” but does not say research time will be protected regardless of receipt of this award.

Study Timeline:

Strengths

- Enrollment and study completion detailed in Table 5 with Aims 1 and 2 completed in first two years.

Weaknesses

- Analysis appears to be complete by year 5.

Budget and Period of Support:

- But placed everything into "other" and really should have divided into proper categories like travel, publication etc.

CRITIQUE 3:

Candidate: 4
Career Development Plan/Career Goals /Plan to Provide Mentoring: 3
Research Plan: 4
Mentor(s), Co-Mentor(s), Consultant(s), Collaborator(s): 1
Environment Commitment to the Candidate: 1

Overall Impact:
The proposal titled “The Use of Mobile Health Technology and Behavioral Economics to Encourage Adherence to Statins in Adolescents with Familial Hypercholesterolemia” looks to leverage the mhealth technology for the problem of medical adherence in teens with a chronic illness. The candidate with an economics background proposes to incorporate novel tech in this approach and to gather information using mixed methodology research. This Resubmission does address many of the concerns in the initial submission, although the resubmit method using [ ] is more difficult to interpret for this reviewer.

1. Candidate:
Strengths

- Economics background coupled with a training in preventive cardiology at one of the largest pediatric cardiology centers.
- Prior MPH is an asset for this proposal.
- Has continued to publish and has been productive since the initial submission and now has a faculty position with protected research time.
- Well supported by senior divisional faculty.

Weaknesses

- Junior faculty but with burgeoning experience in the field of preventive cardiology and healthcare economics.
- No other smaller grant awards other than being investigator on institutional T32.
- Limited experience in running a long-term study.

2. Career Development Plan/Career Goals & Objectives:
Strengths

- The CDP highlights the five year plan with the appropriate coursework and regular meetings with mentors and advisory committees.
- Course work details have been included.
- Goals for each year are well described in the proposal.
- Some lack of clarity as to why Aim needs to encompass the entire 5 year period and how that overlap will influence Aim 3.

Weaknesses

- No major deficiencies noted. Meetings with Dr. Doctor not well described in the CDP plans.

3. Research Plan:

Strengths

- The aims of the study are well described with the novel use of mhealth tech for Aims 1 and 3 and the mixed methodology focus groups for Aim 2. This is an important study to determine way to influence medical non-adherence or to understand interventions better for this group.

Weaknesses

- Concerns around timeline as mentioned above.
- Unclear how study will handle participants that enroll and then fail to participate any meaningful data (i.e. Some data is expected and nonadherence is an outcome of this study, but no data may not add much to the desired aims/objectives).

4. Mentor(s), Co-Mentor(s), Consultant(s), Collaborator(s):

Strengths

- Excellent mentorship group with divisional support for time, salary, and resources.
- Consultants and collaborators that are experts in the field of preventive cardiology and mhealth.

Weaknesses

- None

5. Environment and Institutional Commitment to the Candidate:

Strengths

- Excellent institutional resources and support for the candidate.

Weaknesses

- The institutional make up and “annual report” type of information is unnecessary especially with regards to the number of operations, etc. This is not relevant to the study proposal.

Study Timeline:

Strengths

- Appropriate planned study with regards data collection and recruitment.

Weaknesses

- As mentioned above.

Resubmission:

- Overall the resubmission does address many of the comments from the initial application. ongoing concerns for the study recruitment for a rare disease, but the center is able to justify the volumes based on the overall clinical experience. The incorporation of other chronic illnesses helps with this as well. There is significant innovation using mhealth technology in this realm. Candidate's lack of experience in running a similar clinical trial is of concern.

Budget and Period of Support:

- The description of the budget and the budget forms do not match up easily. Much of the budget in the text is entered in the indirect cost with no entry for the travel for example. In addition, in salary support, this was not entered for the research assistant that is in the descriptive budget.
